# Arthrogryposis Multiplex Congenita Discovered at Birth: A Case Report

**DOI:** 10.7759/cureus.82225

**Published:** 2025-04-14

**Authors:** Chaymae Yechouti, Oulfa Asbik, Anass Ayyad, Sahar Messaoudi, Rim Amrani

**Affiliations:** 1 Department of Neonatology and Neonatal Resuscitation, Mohammed VI University Hospital, Oujda, MAR; 2 Mother and Child Health Laboratory, Faculty of Medicine and Pharmacy, Mohammed First University, Oujda, MAR

**Keywords:** congenita, dysmorphic, genetic etiology, newborn, orthopedic rehabilitation

## Abstract

Arthrogryposis multiplex congenita (AMC) is a rare condition characterized by multiple joint contractures present at birth. It results from fetal akinesia, which disrupts joint development and is accompanied by muscle weakness and fibrosis. Its etiology is heterogeneous.

We report the case of a female newborn. The mother, a 27-year-old woman (G2P2) with no significant medical history, carried the pregnancy to term with a cephalic fetal presentation. Delivery, performed via cesarean section, resulted in a neonate in a state of apparent stillbirth, requiring resuscitation measures. The Apgar scores were 5 at five minutes and 8 at 10 minutes.

The newborn was admitted to the neonatal intensive care unit (NICU) for the management of a polymalformative syndrome associated with neonatal respiratory distress, leading to the suspicion of arthrogryposis. This prompted a karyotype analysis to investigate possible genetic mutations. Unfortunately, the clinical course was fatal.

AMC typically presents with deformities affecting all four limbs, including muscle hypotrophy, radial deviation of the wrists, bilateral hip dislocation, flexion contractures of the knees, and calcaneovalgus foot deformity. Additionally, affected infants may exhibit hypotonia, neurological hearing loss, and global developmental delay.

Currently, no specific treatment exists for AMC. Management is palliative, primarily focusing on rehabilitation, orthotic support, and symptomatic care to improve quality of life.

## Introduction

Arthrogryposis multiplex congenita (AMC) is a rare congenital disorder characterized by multiple joint contractures present at birth, with an estimated prevalence of one in 3,000-5,000 live births [1]. It is often associated with fetal akinesia, a lack of fetal movement that disrupts normal joint development, leading to muscle weakness and fibrosis. Its etiology is highly heterogeneous, involving more than 400 associated disorders, many of which are linked to genetic abnormalities [1]. AMC is typically an autosomal recessive disorder with a rapidly fatal course during the neonatal period [2].

Recent advancements in prenatal ultrasound have significantly improved the early detection of AMC, enabling more accurate prenatal counseling for affected families. While postnatal rehabilitation plays a crucial role in disease management, disabilities often persist. The prognosis largely depends on the timing of joint contracture onset during gestation. An effective approach to managing AMC requires a highly individualized, multidisciplinary strategy tailored to each patient's specific needs and underlying causes [1].

## Case presentation

We report the case of a female newborn from a first-degree consanguineous marriage and a poorly monitored pregnancy. The mother, a 27-year-old woman (G2P2) with no significant medical history, carried the pregnancy to term with a cephalic fetal presentation. Delivery, performed by cesarean section, resulted in a neonate in a state of apparent stillbirth, requiring resuscitation measures. The Apgar score was 5 at five minutes and 8 at 10 minutes.

The newborn was admitted to the neonatal intensive care unit (NICU) for the management of a polymalformative syndrome associated with neonatal respiratory distress, marked by nasal flaring and subcostal and suprasternal retractions. Clinical examination revealed pink skin with peripheral cyanosis, generalized hypotonia, and an absent sucking reflex, necessitating the placement of a nasogastric tube. Assessment of other primitive reflexes was challenging due to the severity of the condition.

**Figure 1:**
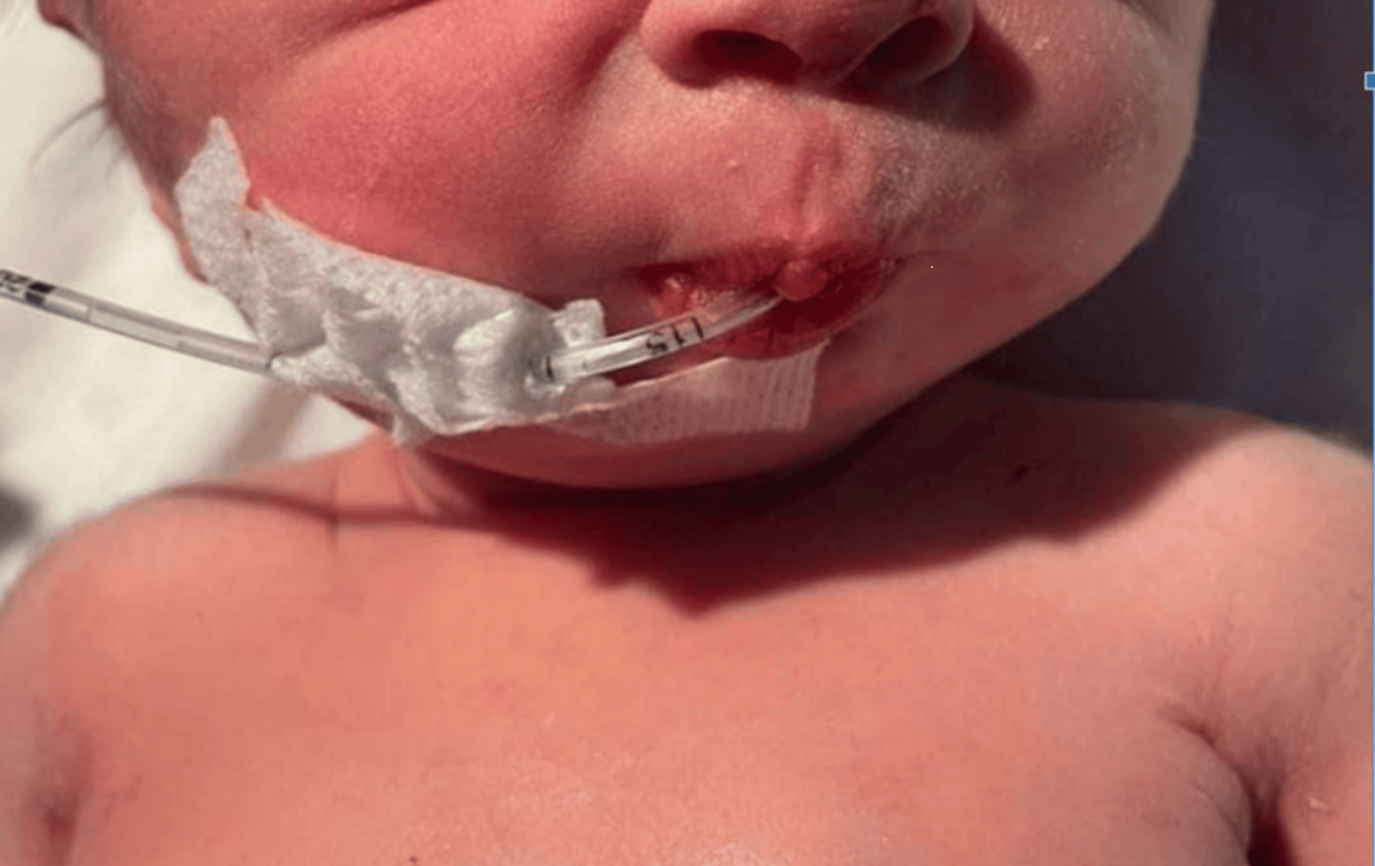
Dysmorphic facies

**Figure 2:**
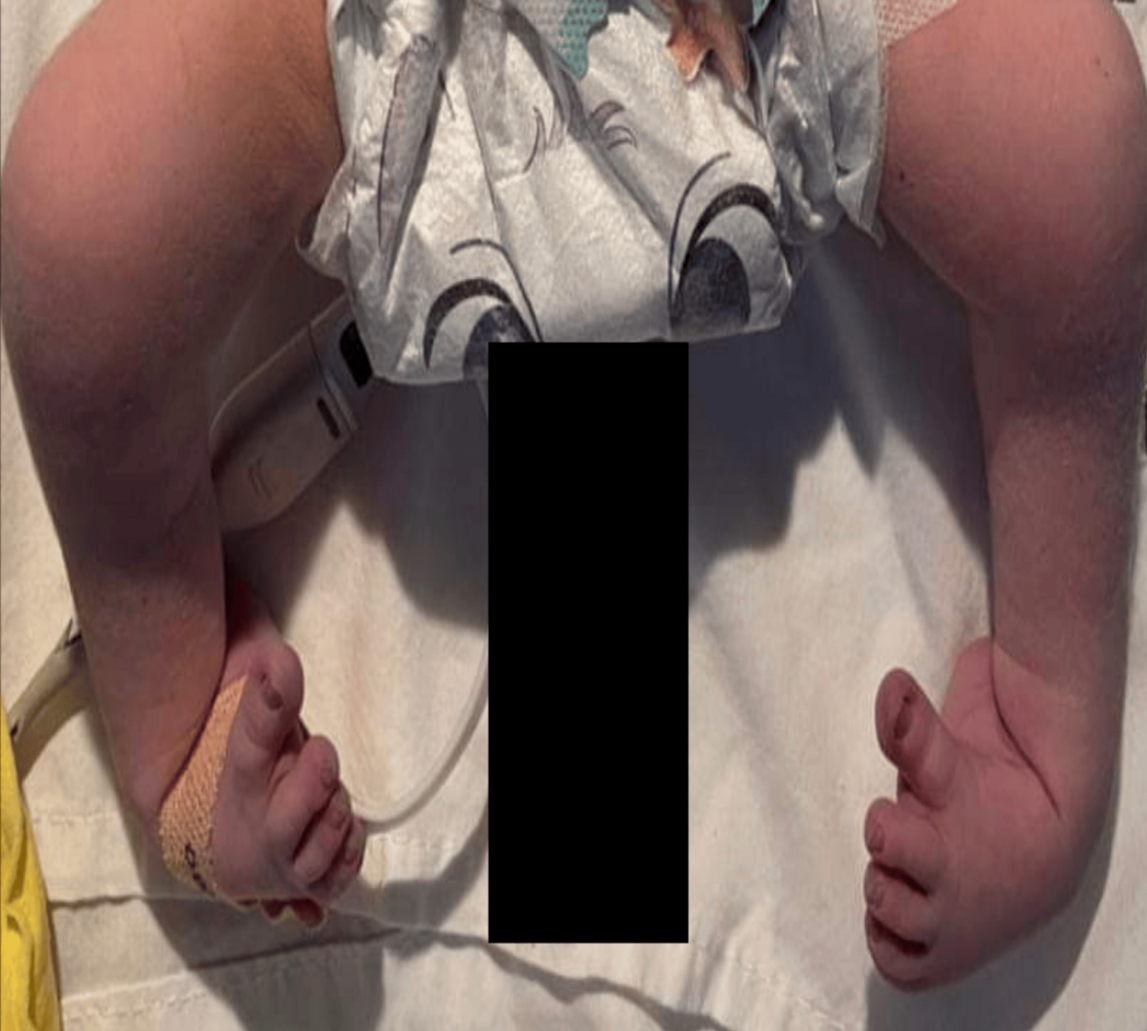
Deformities of both lower limbs

**Figure 3:**
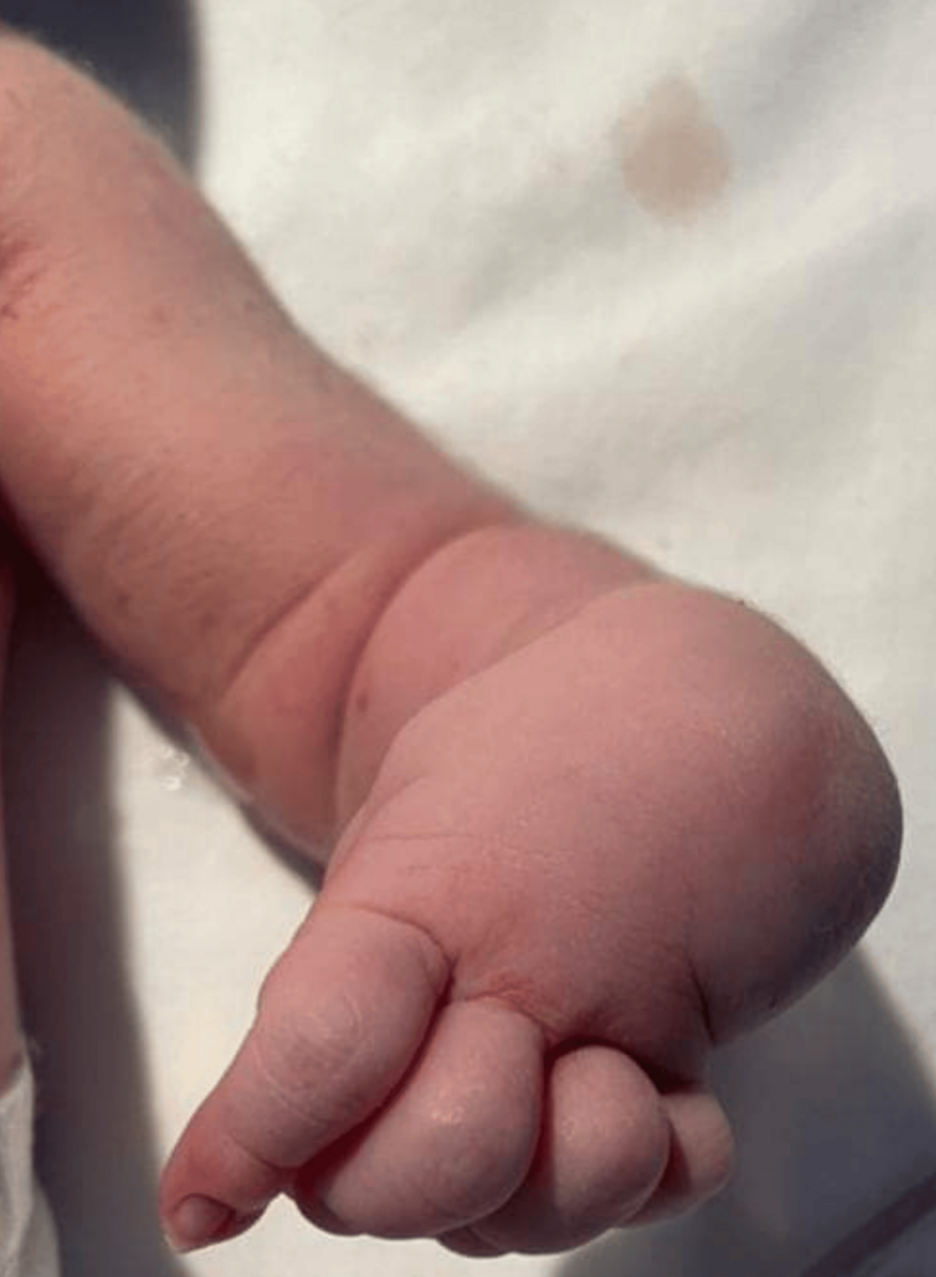
Clubhand and fingers flexed at the proximal interphalangeal joint

**Figure 4:**
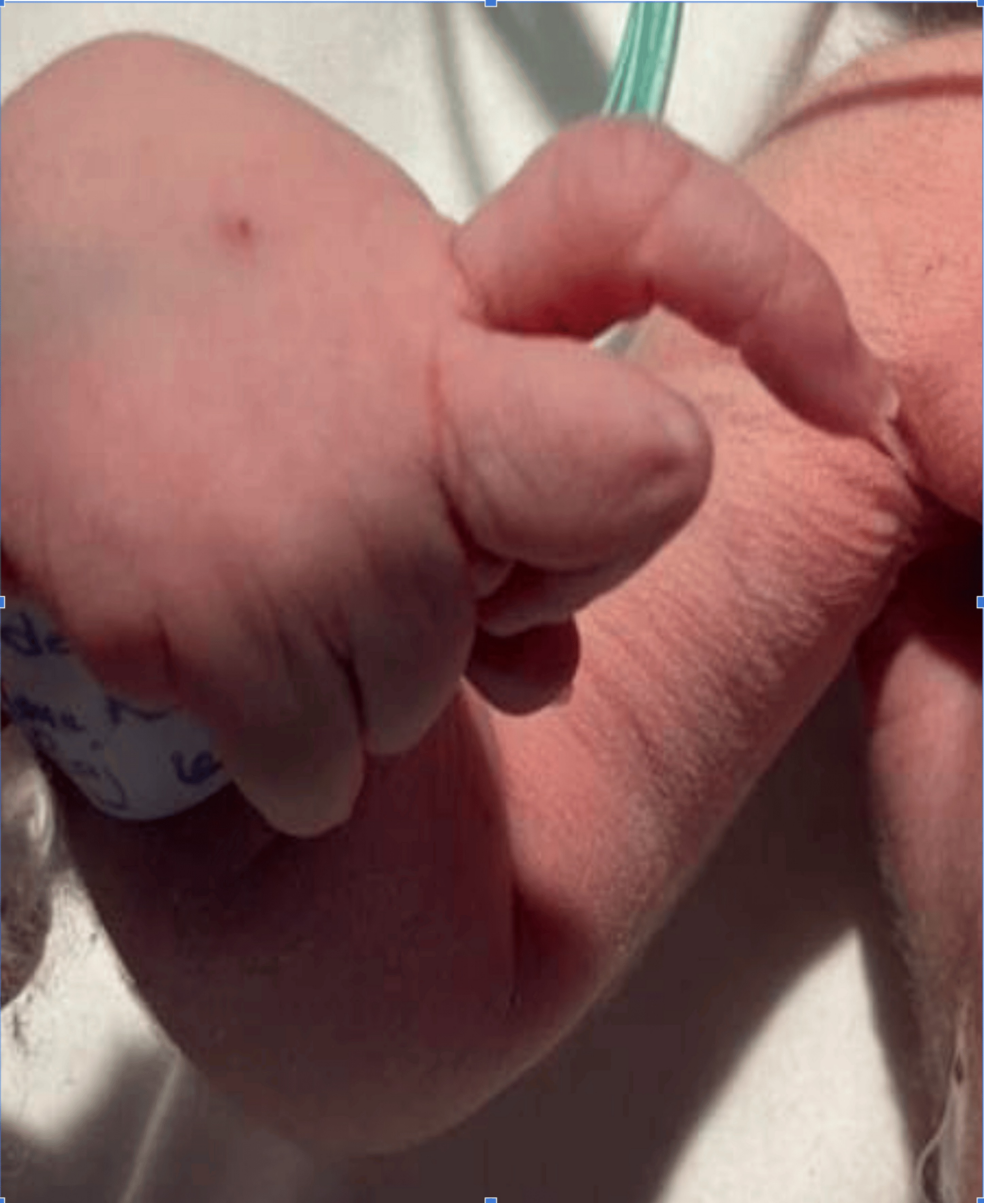
Wrist in flexion and ulnar deviation and thumb in flexion-adduction

The weight, length, and head circumference were within normal parameters (birth weight: 3.2 kg; length: 47 cm; head circumference: 33 cm). Clinical examination revealed a dysmorphic facies characterized by retrognathia (Figure 1), along with the involvement of all four limbs. This included bilateral hip dislocation, knees fixed in flexion, and bilateral talipes equinovarus (Figure 2). The hands showed a clubbed appearance, with wrists in flexion and ulnar deviation, thumbs in flexion-adduction, and other fingers flexed at the proximal interphalangeal joints (Figures 3-4). Pulmonary examination revealed crackles and congenital stridor. Cardiovascular examination showed no abnormalities.

**Figure 5:**
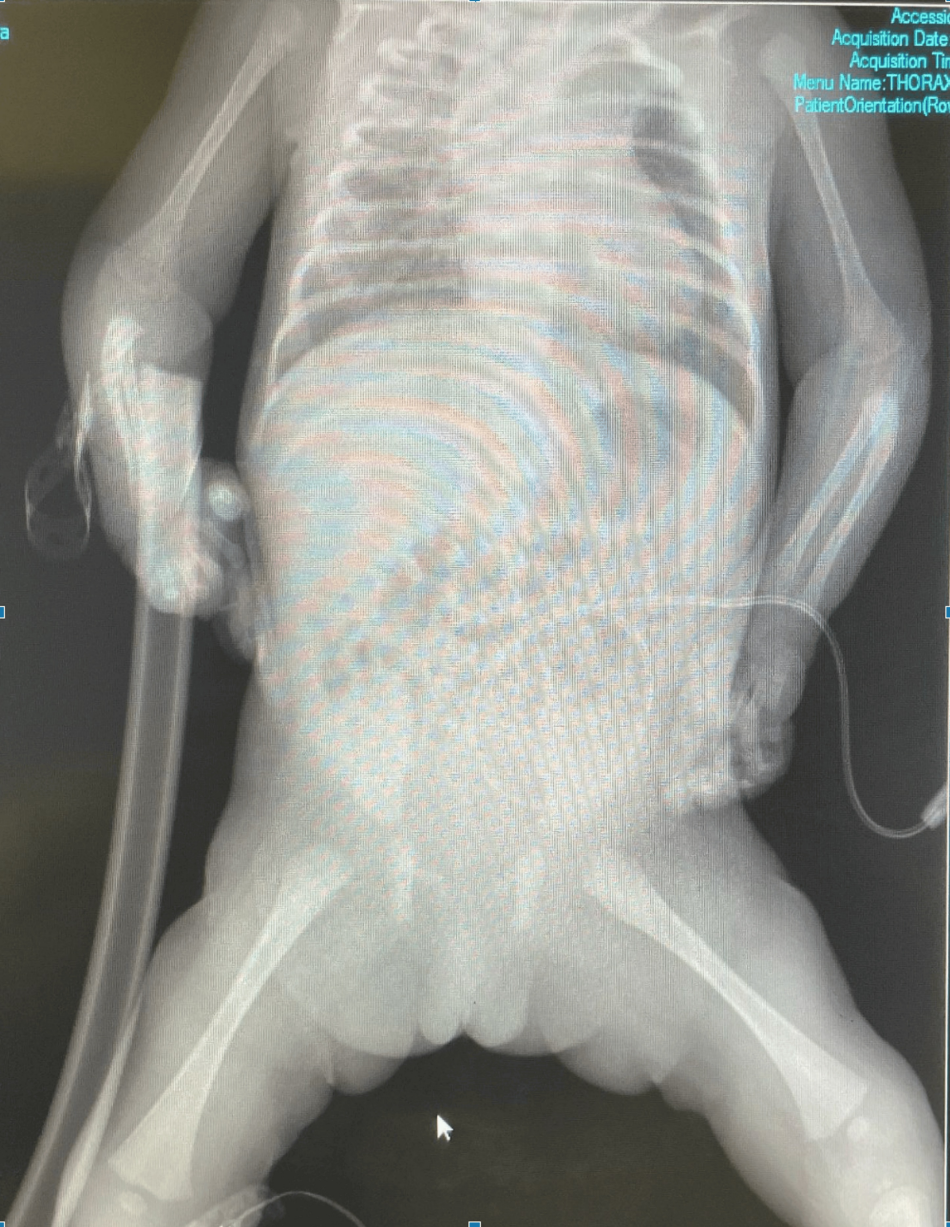
Skeletal radiograph of our patient

**Figure 6:**
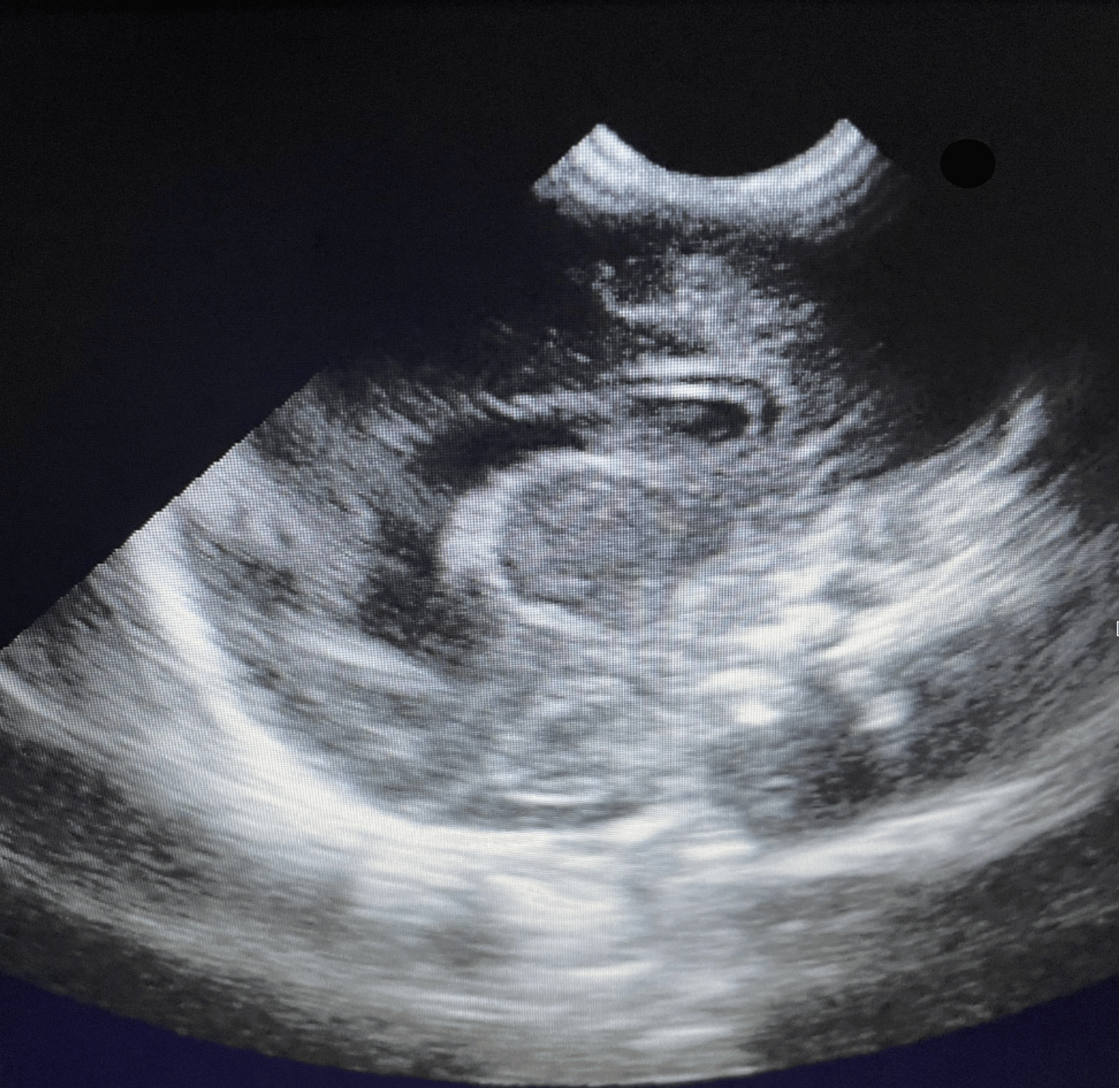
Normal echocardiography of our patient

In light of this clinical presentation, arthrogryposis was suspected, prompting the performance of a karyotype analysis to investigate potential genetic mutations. The results were consistent with a diagnosis of arthrogryposis. A thorough malformative workup was also initiated. Imaging studies, including skeletal radiographs, echocardiography, transfontanellar ultrasound, and abdominopelvic ultrasound, yielded normal results (Figures 5-6). A nasofibroscopy revealed type 2 laryngomalacia.

Despite initial assisted ventilation, the patient's respiratory distress worsened, necessitating intubation. However, the clinical course ultimately proved fatal.

## Discussion

The term "arthrogryposis" means "joint stiffness" [3]. It is a syndrome present at birth, encompassing various conditions that share the common feature of multiple joint contractures, either isolated or associated with visceral or neurological abnormalities. Its prevalence is estimated to range from one in 3,000 to one in 5,100 live births [1].

Recent advancements in molecular genetics have enabled the identification of genetic mutations as well as certain modes of inheritance as the etiology of specific forms of distal arthrogryposis.

First described in 1973 by Christian Nezelaf, this condition is linked to a defect in vacuolar protein sorting 33 homologue B (VPS33B) or VPS33B-interacting protein apical-basolateral polarity regulator (VIPAR), which follows an autosomal recessive mode of inheritance. These two proteins play a critical role in the maturation of late endosomes [4].

The condition is predominantly characterized by deformities affecting all four limbs, although, in some cases, only the lower limbs are involved; isolated involvement of the upper limbs is exceptionally rare. Among foot deformities, the most prevalent is an irreducible congenital talipes equinovarus (clubfoot), typically associated with minimal or absent muscle activity. Other potential presentations include pure equinus foot deformity or a convex foot (rocker-bottom foot), reflecting varying degrees of musculoskeletal involvement. The knees are most commonly fixed in a flexed position; however, they may occasionally present as fully extended or even in hyperextension (genu recurvatum) [1]. The hips are typically positioned in flexion-abduction-external rotation when the femoral heads remain within the acetabulum. In cases of hip dislocation, the affected limb adopts a flexion-adduction posture. Hip involvement, whether associated with dislocation or not, has been documented in approximately 80% of patients diagnosed with arthrogryposis [2].

The shoulder is frequently immobilized in adduction and internal rotation, resulting in significantly reduced mobility. Elbow contractures are common, with the joint fixed in either flexion or extension, while the forearm typically exhibits a pronated posture. The wrists are generally deformed in flexion with ulnar deviation, and the thumb is positioned in flexion-adduction. The remaining fingers often display flexion deformities at the proximal interphalangeal joints, mirroring the presentation observed in our patient. These joint deformities are consistent with the characteristic musculoskeletal findings in arthrogryposis, reflecting underlying fetal akinesia and impaired muscle development [4].

Other anomalies have also been reported, including cranial ossification defects, hip dislocation, talus foot deformity, rigid kyphosis, ichthyosis, a small anterior fontanelle, a simian palmar crease, and cryptorchidism. Additionally, infants exhibit hypotonia, sensorineural hearing loss, and global developmental delay. Magnetic resonance imaging (MRI) may reveal hypoplasia or the absence of the corpus callosum [4].

Vascular involvement of the fetal neuromuscular structures, even if transient, can lead to fetal akinesia and joint contractures [5]. It is also believed that vascular changes play a significant role in the development of abdominal wall malformations, such as gastroschisis, intestinal atresia, and partial or complete absence of finger phalanges.

Arthrogryposis results from neurogenic muscle atrophy. In addition, fractures and osteopenia are common and are secondary to the impaired reabsorption of phosphate and calcium in the proximal renal tubule. A dysfunction of proximal tubular functions, also known as Fanconi syndrome, is often associated with this condition. This tubular disorder manifests with renal tubular acidosis, nephrogenic diabetes insipidus, glycosuria, aminoaciduria, and phosphaturia, which are present in the majority of patients with this syndrome [6]. Renal ultrasound may reveal nephrocalcinosis or dysplastic kidneys that are smaller in size. In our patient's case, the renal ultrasound revealed kidneys of normal size without any apparent parenchymal lesions.

Orthotic devices are used to maintain the joint range of motion achieved through rehabilitation. Correctional casts are helpful for addressing deformities, particularly at the feet and knees. For the upper limbs, the treatment goal is to enhance the ability to perform daily activities, such as feeding, personal hygiene, and ambulation, if necessary, with the aid of crutches or a wheelchair.

There is no curative treatment for AMC. The management is primarily palliative, focusing on symptom alleviation and supportive care. This includes interventions such as increased fluid intake to prevent dehydration, administration of ursodeoxycholic acid to protect liver function, and supplementation of fat-soluble vitamins (A, D, E, K), calcium, L-thyroxine, and phosphate [7]. Due to the rapidly fatal nature of the disease, aggressive orthopedic procedures are generally discouraged, as they do not significantly improve outcomes. Most affected children typically pass away within the first year of life, often due to complications such as recurrent infections, severe dehydration, or major hemorrhage.

## Conclusions

In low-resource settings, such as ours, the lack of comprehensive prenatal screening programs significantly limits the early detection of congenital anomalies, including AMC. This diagnostic delay increases the complexity of neonatal management, often necessitating a more intensive multidisciplinary approach postnatally. The situation further underscores the imperative role of genetic counseling, which is essential for risk stratification, recurrence estimation, and facilitating early detection in subsequent pregnancies.

Given that AMC results from irreversible neuromuscular dysfunction leading to in utero joint contractures, curative treatment remains unavailable. Thus, therapeutic interventions primarily focus on optimizing functional mobility, enhancing joint range of motion, and improving upper limb dexterity to maximize functional independence. Early and intensive physiotherapeutic interventions, orthotic devices, and, in selected cases, surgical correction of severe contractures constitute the mainstay of management. The goal is to enable ambulation, optimize grasp function, and promote activities of daily living (ADLs), thereby facilitating social integration and participation in mainstream education.

The family unit plays a pivotal role in the long-term care of affected children, providing continuous physical, emotional, and logistical support. Psychosocial counseling is crucial in equipping caregivers with coping strategies, given the lifelong rehabilitative needs associated with AMC. Parental education should emphasize early intervention strategies, adherence to physiotherapy regimens, and the importance of multidisciplinary follow-up to ensure optimal developmental outcomes. A structured support system, integrating pediatric orthopedics, neurology, physiotherapy, and psychology, is essential for improving both quality of life and prognosis in affected individuals.
